# Influence of gene expression on survival of clear cell renal cell carcinoma

**DOI:** 10.1002/cam4.3475

**Published:** 2020-09-28

**Authors:** Anders Berglund, Ernest K. Amankwah, Young‐Chul Kim, Philippe E Spiess, Wade J. Sexton, Brandon Manley, Hyun Y. Park, Liang Wang, Jad Chahoud, Ratna Chakrabarti, Chang D. Yeo, Hung N. Luu, Giuliano D. Pietro, Alexander Parker, Jong Y. Park

**Affiliations:** ^1^ Department of Biostatistics and Bioinformatics Moffitt Cancer Center and Research Institute Tampa FL USA; ^2^ Department of Oncology Johns Hopkins University School of Medicine Baltimore MD USA; ^3^ Cancer and Blood Disorders Institute Johns Hopkins All Children’s Hospital Saint Petersburg FL USA; ^4^ Department of Biostatistics Moffitt Cancer Center and Research Institute Tampa FL USA; ^5^ Department of Genitourinary Oncology Moffitt Cancer Center and Research Institute Tampa FL USA; ^6^ Department of Integrated Mathematical Oncology Moffitt Cancer Center and Research Institute Tampa FL USA; ^7^ Department of Cancer Epidemiology Moffitt Cancer Center and Research Institute Tampa FL USA; ^8^ Department of Tumor Biology Moffitt Cancer Center and Research Institute Tampa FL USA; ^9^ Burnett School of Biomedical Sciences University of Central Florida Orlando FL USA; ^10^ Division of Pulmonology Department of Internal Medicine College of Medicine The Catholic University of Korea Seoul Korea; ^11^ Division of Cancer Control and Population Sciences UPMC Hillman Cancer Center University of Pittsburgh Medical Center Pittsburgh PA USA; ^12^ Department of Epidemiology Graduate School of Public Health University of Pittsburgh Pittsburgh PA USA; ^13^ Department of Pharmacy Universidade Federal de Sergipe Sao Cristovao Brazil; ^14^ University of Florida College of Medicine Jacksonville FL USA

**Keywords:** biomarkers, clear cell renal cell carcinoma, gene expression, survival

## Abstract

Approximately 10%‐20% of patients with clinically localized clear cell renal cell carcinoma (ccRCC) at time of surgery will subsequently experience metastatic progression. Although considerable progression was seen in the systemic treatment of metastatic ccRCC in last 20 years, once ccRCC spreads beyond the confines of the kidney, 5‐year survival is less than 10%. Therefore, significant clinical advances are urgently needed to improve overall survival and patient care to manage the growing number of patients with localized ccRCC. We comprehensively evaluated expression of 388 candidate genes related with survival of ccRCC by using TCGA RNAseq (*n* = 515), Total Cancer Care (TCC) expression array data (*n* = 298), and a well characterized Moffitt RCC cohort (*n* = 248). We initially evaluated all 388 genes for association with overall survival using TCGA and TCC data. Eighty‐one genes were selected for further analysis and tested on Moffitt RCC cohort using NanoString expression analysis. Expression of nine genes (AURKA, AURKB, BIRC5, CCNE1, MK167, MMP9, PLOD2, SAA1, and TOP2A) was validated as being associated with poor survival. Survival prognostic models showed that expression of the nine genes and clinical factors predicted the survival in ccRCC patients with AUC value: 0.776, 0.821 and 0.873 for TCGA, TCC and Moffitt data set, respectively. Some of these genes have not been previously implicated in ccRCC survival and thus potentially offer insight into novel therapeutic targets. Future studies are warranted to validate these identified genes, determine their biological mechanisms and evaluate their therapeutic potential in preclinical studies.

## Graphical abstract

Our study identified nine genes as prognostic biomarkers of ccRCC survival. Some of these genes have not been previously implicated in ccRCC survival and thus potentially offer insight into novel therapeutic targets.

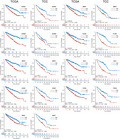

## 1 INTRODUCTION

Renal cell carcinoma (RCC) is one of the most common renal malignancies, with an estimated 73,750 new cases and 14,830 deaths in US in 2020.^1^ Recent studies showed that incidence and mortality rates of RCC are increasing in the United States.^2^ These increased rates may, in part, be due to increasing obesity rates and incidental detection during increased abdominal imaging for nonspecific reasons.^2^, ^3^ Interestingly, obesity also provides an improved survival.^4^ The majority of RCC subtypes are classified as clear cell renal cell carcinoma (ccRCC), which account for almost 70‐75% of all RCCs.^5^ Cancer‐specific survival rate at 5 years for ccRCC patients is 68.9% and ccRCC has a poorer prognosis compared with other RCC such as papillary and chromophobe RCC (*p* < 0.001).^6^, ^7^

The standard of care for localized ccRCC remains surgical excision, and if detected early, ccRCC patients can be cured by surgery. However, about 10%‐20% of ccRCC patients develop metastasis or recurrence following surgical treatment and ultimately die.^7^, ^8^ Although considerable progression was seen in the systemic treatment of metastatic ccRCC in last 20 years, once ccRCC spreads beyond the confines of the kidney, 5‐year survival is less than 10%. Therefore, the identification of reliable biomarkers for ccRCC progression is greatly needed. We and others reported candidate biomarkers, such as long non‐coding RNAs,^9^ gene expression signatures,^10^, ^11^, ^12^, ^13^, ^14^, ^15^ epigenetics^16^ for ccRCC progression and/or survival.^17^, ^18^, ^19^ However, there is currently no clinically accepted molecular biomarker for ccRCC progression.

In this study, we determined the potential expression signature of 388 candidate genes in predicting survival in patients with ccRCC. The association between the expression of candidate genes and overall survival in ccRCC patients was first evaluated in a discovery phase comprising two independent datasets: a cohort of 515 ccRCC patients from The Cancer Genome Atlas (TCGA) and 298 patients from the Total Cancer Care (TCC) data from Moffitt Cancer Center. Eighty‐one genes identified from the discovery dataset were further evaluated as independent predictors of ccRCC survival specifically in a different cohort of 248 ccRCC cases from Moffitt Cancer Center.

## 2 MATERIAL AND METHODS

### 2.1 Discovery datasets

#### 2.1.1 The Cancer Genome Atlas (TCGA)

TCGA KIRC RNAseq data were downloaded from https://gdc.cancer.gov/about‐data/publications/pancanatlas and log2 transformed. Overall Survival (OS) for the TCGA KIRC samples was retrieved from the publication by Liu et al.^20^ This resulted in 515 ccRCC tumor samples with overall survival (OS) data (Table 1). Methylation data was downloaded as RAW IDAT files and normalized.

**Table 1:** Clinical and pathological characteristics of participants for TCC and Moffitt validation.

	TCGA			TCC			Moffitt		
	Dead (n = 169)	Alive (n = 346)	*p* value	Dead (n = 141)	Alive (n = 157)	*p* value	Short‐term survivors (n = 72)	Long‐term survivors (n = 176)	*p* value
Gender			0.65			0.24			0.045
Female	63 (37%)	122 (35%)		49 (35%)	60 (38%)		16 (22%)	62 (35%)	
Male	106 (63%)	224 (65%)		92 (65%)	97 (62%)		56 (78%)	114 (65%)	
Race			0.04			0.139			0.008
Black	11 (7%)	44 (13%)		3 (2%)	6 (4%)		8 (11%)	5 (3%)	
White	155 (92%)	290 (85%)		137 (97%)	145 (92%)		64(89%)	171 (97%)	
Other	1 (1%)	7 (2)		1 (1%)	6 (4%)				
SEER Stage			<0.0001			<0.0001			0.004
Localized	53 (31%)	254 (73%)		46 (33%)	126 (80%)		38 (53%)	120 (68%)	
Reginal	50 (30%)	76 (22%)		39 (28%)	21 (13%)		21 (29%)	21 (12%)	
Distant met	66 (39%)	16 (5%)		53 (38%)	10 (7%)		13 (18%)	35 (20%)	
Unknown				3 (2%)	0 (0%)				
Stage			<0.0001			<0.0001			<0.0001
1	40 (24%)	212 (61%)		33 (23%)	105 (69%)		5 (7%)	110 (62%)	
2	12 (7%)	42 (12%)		14 (10%)	14 (9%)		9 (12%)	13 (7%)	
3	49 (29%)	74 (21%)		32 (23%)	25 (16%)		40 (56%)	31 (18%)	
4	67 (40%)	16 (5%)		55 (39%)	10 (6%)		18 (25%)	22 (13%)	
Unknown	1 (1%)	2 (1%)		7 (5%)	3 (2%)				
Vital Status			<0.0001			<0.0001			<0.0001
Alive	0 (0%)	346 (100%)		0 (0%)	157 (100%)		0 (0%)	137 (78%)	
Dead	169 (100%)	0 (0%)		141 (100%)	0 (0%)		72 (100%)	39 (22%)	

#### 2.1.2 Total Cancer Care (TCC)

Under the TCC protocol, 298 ccRCC tumor tissue samples were collected from patients treated at the Moffitt Cancer Center in 1991‐2009 (Table 1). Tumor RNA was extracted at the centralized Moffitt Tissue Core. Global gene expression assays were performed using a custom Affymetrix HuRSTA (Affymetrix, Santa Clara, CA) GeneChips (HuRSTA‐2a520709, ~60,607 probesets). The expression profile for each sample was extracted from the IRON [32] normalized and de‐batched TCC gene expression database, managed by the Cancer informatics Core (CIC). CIC performs strict quality control and pre‐processing of ccRCC samples, including normalization and removal of RNA‐quality dependent batch effect. Sequence based gene annotation of all probesets on the HuRSTA chip was also obtained from CIC.

### 2.2 Validation dataset

#### 2.2.1 Moffitt RCC cohort

Patients in the validation dataset were 248 ccRCC patients who were surgically treated at the Moffitt Cancer Center between 1992 and 2009 (Table 1). We selected 72 short‐term survivors (<2 years survival after treatment) and 176 long‐term survivors (minimum 5 years survival after treatment). A pathologist (SD) carried out a blinded comprehensive review of all primary tumors to confirm histological subtype (1997 AJCC/UICC classification), tumor stage, 2012 ISUP tumor grade, tumor size, and coagulative tumor necrosis. Representative formalin‐fixed paraffin embedded (FFPE) block with the highest grade was chosen from each resected tumor and tumor region was demarcated for histologic macro‐dissection, which was performed on 10 μm sections. Total RNA was extracted from FFPE tissue sections using the AllPrep DNA/RNA FFPE kit reagents (Qiagen) following the vendor's standard protocols. RNA integrity was assessed via the 260/280 ratio using nanodrop. Gene expression profiling was performed using NanoString platform as described below. Medical records were abstracted and clinical data including age at diagnosis, stage, tumor grade. and metastatic tissue site were recorded. This study was approved by the Moffitt Institutional Review Board.

### 2.3 NanoString platform for gene expression:

The NanoString platform was used to quantify gene expression of genes selected for follow‐up analysis. *HADHA*, *MAEA RBM4*, and *TRIM39* were used as house‐keeping genes. Two hundred nanograms of total RNA from each sample was used for the expression according to the manufacturer's instructions. We determined background hybridization using spiked‐in negative controls. Signals were considered to be below the limits of detection if they were lower than two standard deviations above the mean background. Gene expression was quantified and normalized (positive control normalization and housekeeping gene normalization using geometric mean) using NanoString nSolverTM 4.0 software. Expression values were log2 transformed and exported to MATLAB R2020b software for further analysis.

### 2.4 Gene selection

Candidate ccRCC‐related genes were selected based on previous published literature.,^9^, ^10^, ^11^, ^12^, ^13^, ^14^, ^15^, ^16^, ^17^, ^18^, ^19^ including our review article ^19^ and pathways described in the Cancer Genome Anatomy project. A total of 388 candidate genes were evaluated in the discovery sets. A subset of 81 genes were selected from the discovery sets for further evaluation in the validation set based on the reproducibility in the two discovery datasets (*n* = 52 genes), prognostic value, and biological relevance (*n* = 29 genes, e.g. known function in ccRCC or other cancers) (Table S2).

### 2.5 Statistical analysis

Participants’ demographic and clinical characteristics were summarized using descriptive statistics, counts, and percentages for categorical variables and means and standard deviations or median and ranges for numeric variables. All genes in the discovery datasets were tested for association with overall survival using Kaplan‐Meier (K‐M). For the K‐M, the median expression was used as a cutpoint to dichotomize expression. In the validation dataset, we compared differential gene expression (of the identified genes in the discovery datasets) between aggressive and indolent cases of ccRCC using t‐test. P values were used to select candidate genes. Genes with *p* < 0.01 in both sets were considered to be differentially expressed. Univariate and multivariate Cox proportional hazards regression models were sequentially built with genes and clinical prognostic factors (tumor stage and grade) for TCGA and TCC data set and followed by time‐dependent receiver‐operating characteristic curve (ROC) analysis at year 5. Logistic regression was used along with ROC analysis for Moffitt dataset analysis. Area under ROC curve (AUC) were calculated to compare prognostic values of the models.

## 3 RESULTS

### 3.1 Results from Discovery phase using TCGA and TCC data

We screened 388 ccRCC‐related candidate genes for their association with overall survival in two independent discovery datasets. A total of 148 genes were associated with OS using RNA‐seq data of 515 ccRCC patients from the TCGA data portal and 99 genes using Affymetrix data for 298 ccRCC patients from TCC (log rank *p*‐value <0.01). A subset of 81 genes was selected for further evaluation using NanoString in the Moffitt validation set. The selection of these genes was based on overlap between the two discovery datasets (*n* = 52) and manual curation that integrated statistical and biological information (*n* = 29), with function in ccRCC or other cancers were favored known).

### 3.2 Results from validation set, Moffitt cohort using NanoString assay

**Figure 1:**
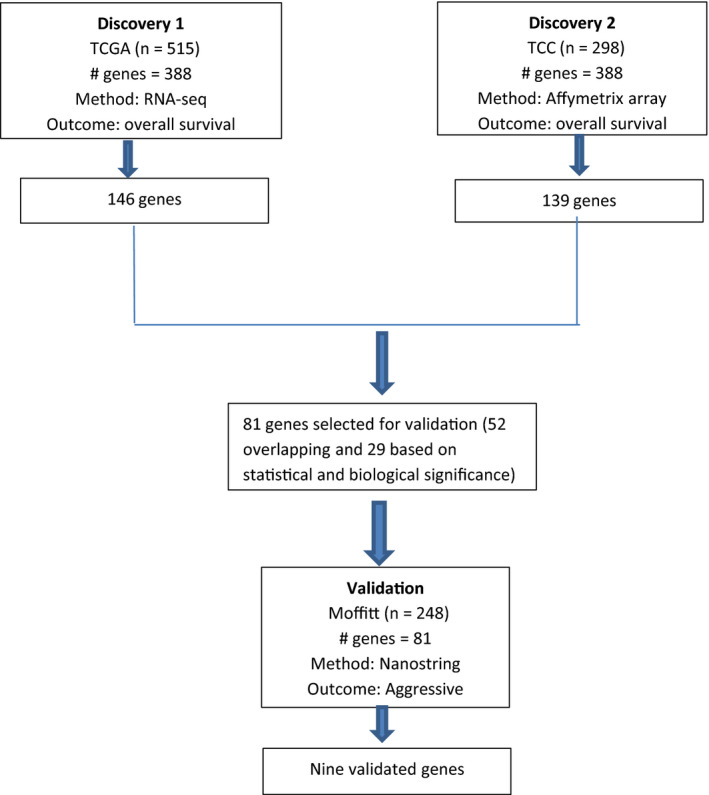
Outline of overall study design. Data from 515 and 298 patients, respectively, were obtained from TCGA and TCC. A COX regression analysis identified genes with expression levels associated with overall survival. Expression levels of 81 genes were further evaluated using NanoString in 248 cases

**Figure 2:**
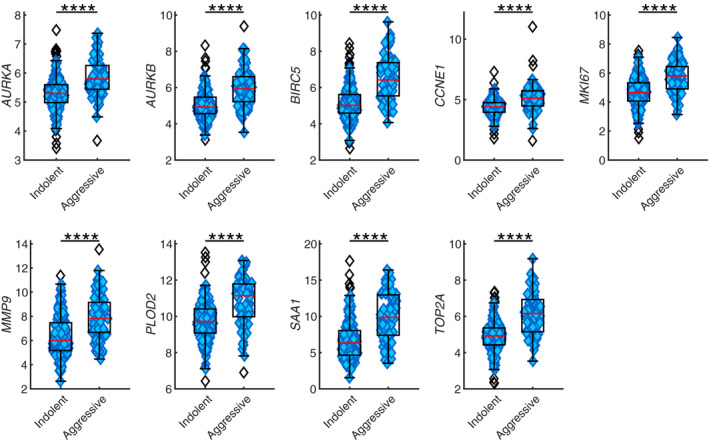
Boxplots of nine genes. Nine genes (*AURKA*, *AURKB*, *BIRC5*, *CCNE1*, *MK167*, *MMP9*, *PLOD2*, *SAA1*, *and TOP2A*) were confirmed based on expression level between short‐ and long‐term survivor cases in validation set. All the nine genes were overexpressed in short‐term survivors (aggressive) compared to long‐term survivors (indolent). ****p < 0.0001

**Figure 3:**
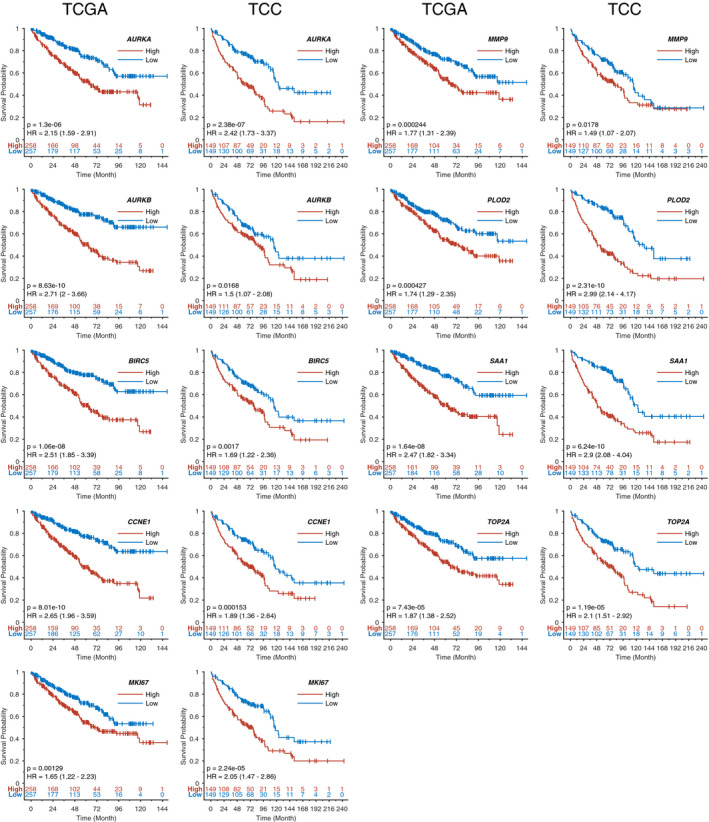
Overexpression of these nine genes was associated with poor overall survival in the TCGA and TCC datasets with hazard ratios (HRs) ranging from 1.49 to 2.99 in discovery set

The demographic and clinical characteristics of participants are in Table 1. The expression of the resulting 81 genes were measured using NanoString on 72 short (<2 years survival after treatment) and 176 long‐term survivors (minimum 5 years survival) (Figure 1). Nine genes (*AURKA*, *AURKB*, *BIRC5*, *CCNE1*, *MK167*, *MMP9*, *PLOD2*, *SAA1*, *and TOP2A*) were confirmed based on expression level between short‐ and long‐term survivor cases (Figure 2). All the nine genes were overexpressed in short survivors compared to long‐term survivors. *SAA1* (Log2 fold change (FC)=3.52, p = 1.01E‐10) was the most highly overexpressed, followed by *MMP9* (FC=1.82, p = 3.82E‐09), *BIRC5* (FC=1.40, p = 2.39E‐14), *PLOD2* (FC=1.42, p = 9.79E‐10), *TOP2A* (FC=1.24, p = 7.32E‐14), *MKI67* (FC=1.13, p = 7.41E‐11), *AURKB* (FC=0.99, p = 2.86E‐11) *CCNE1* (FC=0.71, p = 8.01E‐09), and *AURKA* (FC=0.49, p = 1.90E‐9) (Table 2). Overexpression of these nine genes were associated with poor overall survival in the TCGA and TCC datasets with hazard ratios (HRs) ranging from 1.49‐2.99 (Figure 3). The association between expression of *AURKB*, *BIRC5*, *CCNE1*, *MMP9*, *SAA1*, *TOP2A*, and overall survival was slightly lower in the TCC dataset compared to that of TCGA, while the association was higher in TCC compared to TCGA for *AURKA*, *MKI67*, and *PLOD2*.

**Table 2:** Discovery and Validation of genes associated with survival.

Gene	Location	TCGA		TCC		Moffitt	
		HR	*p* value	HR	*p* value	FC*	*p* value
AURKA	20q13	2.15 (1.59‐2.91)	1.30E−06	2.42 (1.73‐3.37)	2.38E−07	0.49	1.90E−9
AURKB	17p13.1	2.71 (2.00‐3.66)	8.63E−10	1.50 (1.07‐2.08)	1.68E−02	0.99	2.86E−11
BIRC5	17q25	2.51 (1.85‐3.39)	1.06E−08	1.69 (1.22‐2.36)	1.70E−03	1.40	2.39E−14
CCNE1	19q12	2.65 (1.96‐3.59)	8.01E−10	1.89 (1.36‐2.64)	1.53E−04	0.71	8.01E−09
MKI67	10q26.2	1.65 (1.22‐2.23)	1.29E−03	2.05 (1.47‐2.86)	2.24E−05	1.13	7.41E−11
MMP9	20q11.2‐q13.1	1.77 (1.31‐2.39)	2.44E−04	1.49 (1.07‐2.07)	1.78E−02	1.82	3.82E−09
PLOD2	3q24	1.74 (1.29‐2.35)	4.27E−04	2.99 (2.14‐4.17)	2.31E−10	1.42	9.79E−10
SAA1	11p15.1	2.47 (1.82‐3.34)	1.64E−08	2.90 (2.08‐4.04)	6.24E−10	3.52	1.01E−10
TOP2A	17q21‐q22	1.87 (1.38‐2.52)	7.43E−05	2.10 (1.51‐2.92)	1.19E−05	1.24	7.32E−14

* Log 2‐fold change.

### 3.3 Testing the prognostic risk models using ROC analysis

**Figure 4:**
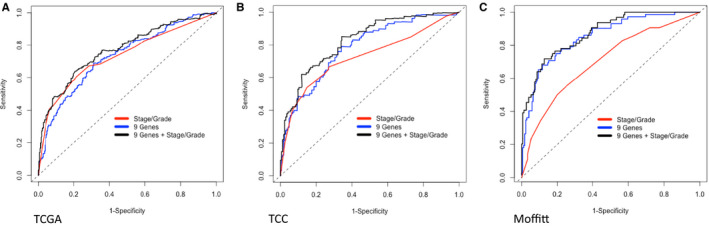
Analysis of survival prognostic risk models: three models (nine genes, stage/grade, and combined) for TCGA, TCC, and Moffitt data. 4A. multivariate Cox regression models showed time‐dependent AUC of 0.731, 0.737, and 0.776 in TCGA. 4B. AUC of 0.783, 0.716, and 0.821 in TCC. 4C. logistic regression model for Moffitt data showed AUC of 0.852, 0.702, and 0.873

Cox regression models with nine genes, or clinical prognostic factors (tumor stage and grade) showed AUC of 0.731, 0.737 in TCGA (Figure 4A), 0.783, 0.716 in TCC data set (Figure 4B). Incorporating 9 genes into clinical factors regression models yielded non‐significantly increased AUC values of 0.776 in TCGA and 0.821 in TCC. On the other hand, combining nine genes with the clinical factors significantly improved AUC from 0.702 to 0.873 in Moffitt validation dataset (Figure 4C; Table S1).

### 3.4 Epigenetic regulation of *SAA1* and *PLOD2*

**Figure 5:**
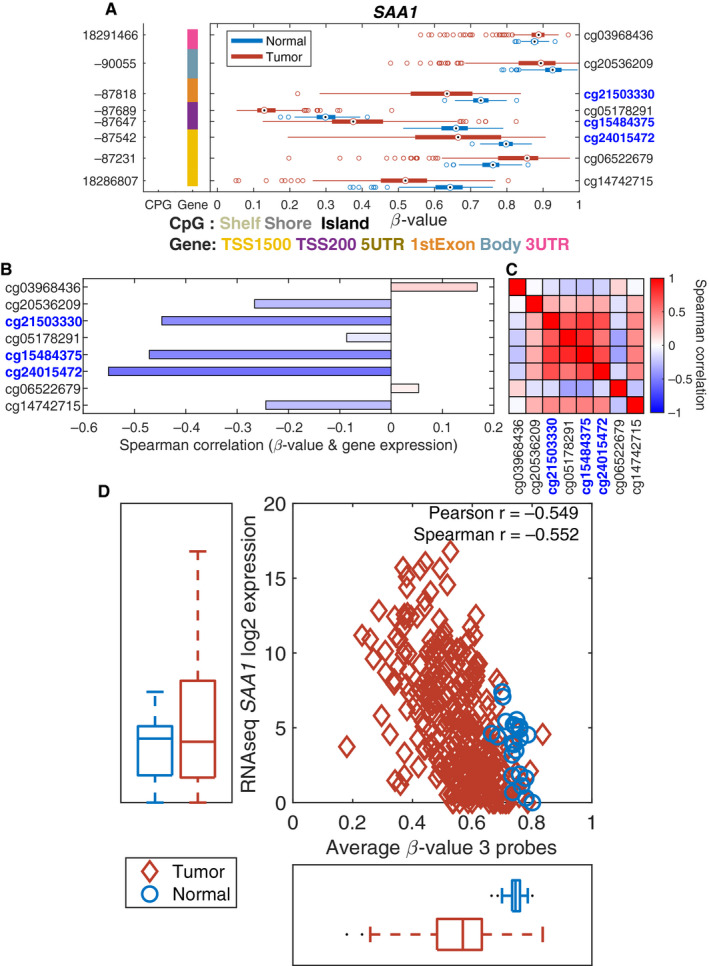
Methylation driven expression of *SAA1*. 5A. A box‐plot for each of the eight CpG‐probes for *SAA1* comparing tumor vs normal samples. 5B. A negative correlation between the methylation and the expression level. 5C. A high degree of correlation between methylation and the expression level. 5D. Hypomethylation of these CpG sites leads to an increased expression

To investigate whether any of these nine genes was epigenetically regulated by methylation, we used the TCGA dataset, combining RNAseq and Illumina 450 K methylation data. Two of the nine genes, *SAA1* and *PLOD2* showed a negative correlation between methylation level and gene expression level, indicating that these genes are regulated by methylation. Figure 5A shows a box‐plot for each of the 8 CpG‐probes for *SAA1* comparing tumor tissue vs normal samples. The data suggested that many tumor samples show hypomethylation compared to normal samples. Three of the probes showed a clear negative correlation between the methylation level and the expression level (Figure 5B) and these probes also showed a high degree of correlation between each other (Figure 5C). The hypo‐methylation of these three probes led to an increased expression (Figure 5D).

**Figure 6:**
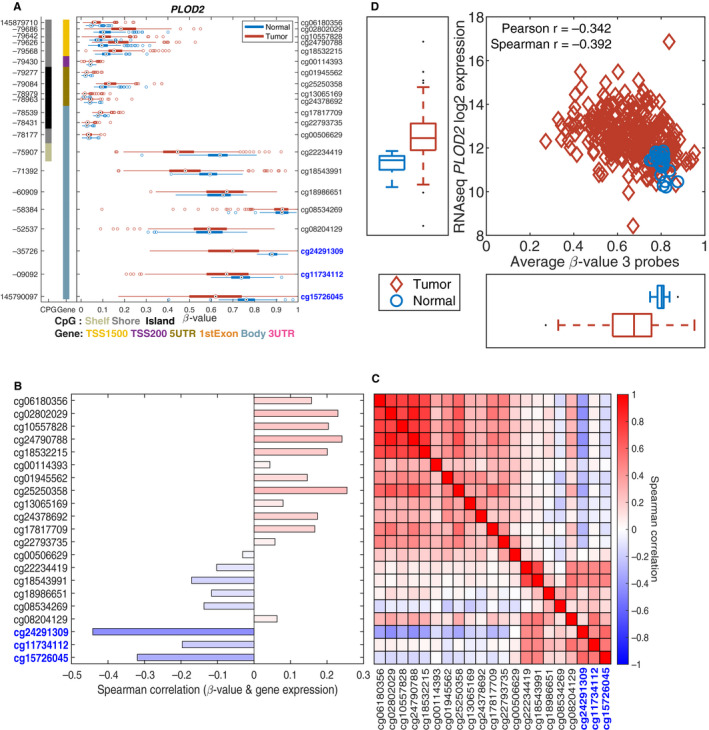
Methylation driven expression of *PLOD2*. 6A. A box‐plot for each of the 21 CpG‐probes for *PLOD2* comparing tumor vs normal samples. 6B. A negative correlation between the methylation and the expression level. 6C. A high degree of correlation between methylation and the expression level. 6D. Hypomethylation of these CpG sites leads to an increased expression

*PLOD2* showed a more complicated methylation profile (Figure 6). There are 21 CpG‐probes available for *PLOD2* (Figure 6A) with three probes being negatively correlated (*r* < −0.3) with expression value (Figure 6B). These three CpG‐probes are also correlated (Figure 6C). The average methylation for these CpG‐probes was negatively correlated with the expression level of *PLOD2* (Figure 6D).

## 4 DISCUSSION

In this study, we used a multi‐stage design to identify genes associated with ccRCC survival. First, we evaluated, in two independent datasets, the association between overall survival and 388 genes identified through literature search. We then selected 81 genes based on the magnitude of the association with outcome, overlap in the two discovery datasets and biological relevance for validation using the NanoString platform in an independent cohort that included 72 short‐term survivors. Differential expression for nine genes (*AURKA*, *AURKB*, *BIRC5*, *CCNE1*, *MKI67*, *MMP9*, *PLOD2*, *SAA1*, *and TOP2A*) was validated on the NanoString platform. Six of these validated genes (*BIRC5*, *MK167*, *MMP9*, *PLOD2*, *and TOP2A*) have previously been implicated ccRCC,^21^, ^22^, ^23^, ^24^, ^25^, ^26^, ^27^, ^28^, ^29^, ^30^, ^31^, ^32^, ^33^, ^34^ while the others have been implicated in other cancers.^35^, ^36^, ^37^, ^38^ The prognostic value of models with nine genes and clinical prognostic factors are 0.776, 0.821, and 0.873 in TCGA, TCC and Moffitt data set, respectively.

BIRC5, Baculoviral IAP Repeat Containing 5, or survivin plays a role in apoptotic cell death. Differential expression is associated with survival in ccRCC.^21^ Survivin expression is increased in ccRCC compared to adjacent normal renal tissues and the expression is positively correlated with pathological grade and clinical stage.^22^ Expression of the survivin protein is associated with ccRCC progression and poor survival.^23^, ^24^

MKI67, marker of proliferation Ki67, is a nuclear protein associated with cellular proliferation. It has also been implicated in poor outcomes for patient undergoing surgery for localized ccRCC,^25^ metastasis of ccRCC,^26^ and recurrence of ccRCC after surgery.^39^ It is also upregulated in ovarian cancer cell lines treated with estradiol or genistein suggesting a role in drug response.^27^

MMP9, Matrix Metallopeptidase 9, plays a role in the breakdown of extracellular matrix during cancer metastasis. It is highly expressed in ccRCC^40^ and implicated in the pathogenesis of ccRCC.^28^ Upregulation of *MMP9* is also associated with migration and invasion^29^ and progression of ccRCC.^30^ *MMP9* was recently included in a 4‐gene prognostic prediction set for predicting the prognosis of ccRCC.^31^

PLOD2, Procollagen‐Lysine, 2‐Oxoglutarate 5‐Dioxygenase 2, is a hypoxia‐induced membrane‐bound homodimeric enzyme that is involved in collagen synthesis and extracellular matrix degradation. *PLOD2* is overexpressed in ccRCC and downregulation significantly inhibits cell migration and invasion.^32^ Overexpression of *PLOD2* is also associated with lymph node metastasis and poor recurrence‐free and overall survival in biliary,^41^ breast,^42^ hepatocellular carcinoma (HCC),^43^ cervical,^44^ lung.^45^, ^46^ gastric,^47^ glioma^48^ and bladder^49^ cancers.

TOP2A, DNA Topoisomerase II Alpha, plays an important role in transcription through controlling and altering topologic states of DNA. It is upregulated in ccRCC and associated with progression and prognosis, especially survival among patients undergoing surgery, with a more prominent prognostic value among patients with low‐risk disease.^30^, ^33^, ^34^ It is also important for the progression of prognostic marker for papillary RCC.^50^

Aurora‐A (AURKA) and Aurora‐B (AURKB) are kinases that play key roles in the regulation of cell‐cycle progression.^51^, ^52^, ^53^ In addition to cell cycle regulation, Aurora‐A is involved in contributing to epithelial‐mesenchymal transition (EMT) and stem‐like properties of cancer cells.^54^ It is implicated in the activation of the mTOR pathway in sarcomatoid RCC.^55^ A recent study showed that it is overexpressed in metastasis compared to primary RCC tumors.^56^ In addition, *AURKA* is involved in the pathogenesis or progression of hepatocarcinoma,^35^ bladder,^36^ breast,^37^ liver,^38^ gastric,^57^ colon,^58^ non‐small cell lung,^59^ and pancreatic^60^ cancers. AURKB modulates drug response in non‐small cell lung,^61^ and breast cancers.^62^ It is implicated in chronic myelocytic leukemia^63^ gastric cancer,^64^ and leukemia.^65^

CCNE1 and SAA1 have not been previously implicated in RCC, but are implicated in other cancers. CCNE1 is a major G1/S phase cyclin. It is associated with aggressive potential in endometrial cancer.^66^, ^67^ Upregulation of *CCNE1* is associated with worse prognosis of ovarian clear cell carcinoma^68^ and treatment resistance and poor outcome in high grade serous ovarian cancer.^69^ Upregulation of *CCNE1* is also observed in aggressive osteosarcoma^70^ and also implicated in cisplatin resistance in bladder cancer.^71^ Differential expression of *CCNE1* is also observed in other cancers including non‐small cell lung cancer (NSCLC),^72^ bladder,^73^ breast^74^ and hepatocellular carcinoma.^75^

Serum Amyloid A1, SAA1, is an apolipoprotein that is highly expressed in response to inflammation and tissue injury. It is overexpressed in glioblastoma (GBM),^76^, ^77^ cervical carcinoma,^78^ NSCLC,^79^ AML,^80^ and gastric cancer^81^ and associated with progression and poor prognosis in these cancers.

Previous studies reported differential expressions of various genes in clinical specimens of ccRCC compared to adjacent uninvolved renal tissues and suggested that these genes may serve as promising ccRCC risk stratification biomarkers.^19^, ^82^ Nuclear HIF expression, elevated expression of Ubiquitin Protein Ligase E3C (UBE3C), reduced expression of phos‐Akt in the nucleus and CAIX and loss of p27 expression are reported as significant independent prognostic factors for poor ccRCC outcomes.^83^, ^84^, ^85^, ^86^, ^87^ A gene signature with five protein markers (Ki‐67, p53, endothelial VEGFR‐1, epithelial VEGFR‐1, and epithelial VEGF‐D) was proposed to predict survival for ccRCC with AUC of 0.838.^88^ *CXCL13* was upregulated in ccRCC tumor tissues and CXCL13 expression was associated with advanced stage and poor prognosis in ccRCC. Therefore, CXCL13 expression was proposed as a diagnostic biomarker for ccRCC with AUC of 0.809.^82^ Receptor tyrosine kinase (*TEK*) plays an important role in angiogenesis and remodeling. Downregulation of *TEK* expression was observed in ccRCC tissues and associated with poor outcome with AUC between 0.637 and 0.839.^89^ Recently, seven differentially expressed autophagy‐related genes (*PRKCQ*, *BID*, *BAG1*, *BIRC5*, *ATG16L2*, *EIF4EBP1*, and *ATG9B*) were included in a recent prognostic survival assessment tool for ccRCC with AUCs of 0.752 and 0.783 for overall and disease free survival, respectively.^90^ Similarly, 10 differentially expressed genes (*AGR3*, *CSF2*, *GAL3ST2*, *IGLL1*, *PLG*, *SAA1*, *SBSN*, *SOX2*, *WFDC13*, and *ZIC2*) were included in a recent prognostic risk assessment tool for ccRCC with AUC of 0.99 without a validation set.^91^ The results from previous studies are not consistent with one from the current study. Potential reasons for inconsistency can be small sample sizes, different racial/ethnic background, potential environmental factors and more importantly, lack of validation set.

Dysregulation of gene expression in ccRCC could be caused by various genomic aberrations, such as methylation. We observed that two of the nine genes *SAA1* and *PLOD2* gene expression is partly regulated by DNA methylation levels in the promoter region. This is similar to what we have seen in our other studies using the TCGA gene expression and methylation data.^92^, ^93^

Strengths of our study include a multistage design that consisted of two discovery independent discovery datasets and a validation dataset; the use of NanoString platform that allows for accurate and reproducible measurement of RNA from FFPE samples; selection of candidate genes based on a thorough literature search^9^, ^10^, ^11^, ^12^, ^13^, ^14^, ^15^, ^16^, ^17^, ^18^, ^19^; and validation of the role of identified genes previously implicated in ccRCC and are potential targets for novel cancer therapies. Indeed, inhibitors of some of these genes are already in early phase I trials.^94^, ^95^, ^96^ We also identified genes that have not been previously identified in ccRCC and represent possible targets for therapy and warrant further evaluation.

A limitation of our study is the selection of the 29 out of the 81 genes for validation based on biological significance. This selection process could potentially introduce selection bias. Thus, candidate gene approach may exclude critical genes for ccRCC survival. In addition, spatial heterogeneity could result in different clonal populations within the same tumor and affect the expression profiling of the evaluated genes in the tumors. Future studies are therefore required to validate our results, use immunohistochemistry to assay the corresponding protein expression, as well demonstrate that therapeutic targeting of the identified genes will result in inhibition of tumor growth. Another limitation is the use of RNA extracted from FFPE tumor tissue. RNA from FFPE may be degraded, but the NanoString platform allows accurate and reproducible measurement of RNA from FFPE samples. The discovery and validation cohorts differed in that the validation cohort was comprised largely of aggressive cases. Furthermore, the gene expression in the discovery datasets was generated on RNA‐seq (for TCGA) and Affymetrix array (for TCC), while the validation study utilized NanoString platform. Despite the differences in the platforms, we identified genes that were consistently differentially expressed in the three different cohorts. In summary, our study identified nine genes as prognostic biomarkers of aggressive ccRCC that are differentially expressed during progression. Some of these genes have not been previously implicated in ccRCC and thus potentially offer insight into novel therapeutic targets. These biomarkers may help to identify aggressive ccRCC patients, who need more intensive treatment. Future studies are warranted to validate the identified genes, determine their biological mechanisms, and evaluate their therapeutic potential in preclinical studies.

## CONFLICT OF INTEREST

No author has COI.

## AUTHORS CONTRIBUTION

Conception or design of the work was done by AB, EKA, YCK, PES, WJS, BM, RC, CDY, HNL, GDP, AP, and JYP. Data collection was done by AB, PES, WJS, BM, HYP, LW, JC, RC, CDY, HNL, and GDP. Data analysis and interpretation were done by AB, EKA, YCK, PES, WJS, BM, LW, JC, RC, CDY, HNL, GDP, AP, and JYP. Drafting the article was done by AB, EKA, YCK, and JYP. Critical revision of the article was done by AB, EKA, YCK, BM, and JYP. Final approval of the version to be published was done by all authors, AB, EKA, YCK, PES, WJS, BM, HYP, LW, JC, RC, CDY, HNL, GDP, AP, and JYP.

## Supporting information

Table S1‐S2

## Data Availability

The data that support the findings of this study are available on request from the corresponding author, JYP.
