# Supplementary material for: Influence of gene expression on survival of clear cell renal cell carcinoma
Source: Cancer Med. 2020 Sep 28;9(22):8662–75. doi: 10.1002/cam4.3475 (PMC7666730; doi:10.1002/cam4.3475)
Supplement: Supplementary file 1 — Table S1‐S2 [file CAM4-9-8662-s001.docx]

**Supplemental data**

**Table 1. C-index values in different models in three data set**

|  |  | **TCGA** | | | | **TCC** | | | | **Moffitt** | | | |
| --- | --- | --- | --- | --- | --- | --- | --- | --- | --- | --- | --- | --- | --- |
| Model | Features in Model | AUC | 95% CI | | | AUC | | 95% CI | | AUC | 95% CI | |  |
| 1 | AURKA | 0.625 | 0.559 | 0.691 | 0.684 | | 0.612 | | 0.755 | 0.744 | 0.675 | 0.813 |  |
| 2 | AURKB | 0.677 | 0.615 | 0.739 | 0.577 | | 0.499 | | 0.655 | 0.770 | 0.705 | 0.836 |  |
| 3 | BIRC5 | 0.679 | 0.617 | 0.741 | 0.582 | | 0.503 | | 0.661 | 0.810 | 0.751 | 0.870 |  |
| 4 | CCNE1 | 0.698 | 0.637 | 0.760 | 0.654 | | 0.581 | | 0.728 | 0.734 | 0.659 | 0.810 |  |
| 5 | MKI67 | 0.603 | 0.536 | 0.671 | 0.646 | | 0.571 | | 0.721 | 0.765 | 0.699 | 0.831 |  |
| 6 | MMP9 | 0.611 | 0.547 | 0.676 | 0.637 | | 0.562 | | 0.712 | 0.739 | 0.673 | 0.806 |  |
| 7 | PLOD2 | 0.594 | 0.528 | 0.660 | 0.728 | | 0.658 | | 0.797 | 0.748 | 0.675 | 0.822 |  |
| 8 | SAA1 | 0.664 | 0.601 | 0.727 | 0.733 | | 0.666 | | 0.800 | 0.763 | 0.695 | 0.830 |  |
| 9 | TOP2A | 0.605 | 0.538 | 0.672 | 0.632 | | 0.555 | | 0.708 | 0.804 | 0.741 | 0.867 |  |
| 10 | All 9 genes | 0.731 | 0.672 | 0.789 | 0.783 | | 0.722 | | 0.843 | 0.852 | 0.801 | 0.904 |  |
| 11 | stage | 0.716 | 0.657 | 0.774 | 0.711 | | 0.645 | | 0.776 | 0.601 | 0.530 | 0.673 |  |
| 12 | grade | 0.671 | 0.612 | 0.730 | 0.620 | | 0.549 | | 0.692 | 0.588 | 0.509 | 0.667 |  |
| 13 | Stage + Grade | 0.737 | 0.678 | 0.797 | 0.716 | | 0.645 | | 0.787 | 0.702 | 0.626 | 0.778 |  |
| 14 | Stage + Grade + All 9 genes | 0.776 | 0.721 | 0.831 | 0.821 | | 0.766 | | 0.876 | 0.873 | 0.824 | 0.921 |  |

**Table 2. Association of gene expression with survival in primary ccRCC tumor (TCGA and TCC).**

| GeneSymbol | Probeset Id | Location | Class | log rank p OS TCGA | log rank p OS TCC |
| --- | --- | --- | --- | --- | --- |
| ABCC1 | merck2-NM_019898_at | 16p13.1 | Overexpression | 1.34E-03 | 3.19E-03 |
| ABCG2 | merck2-BC021281_at | 4q22 | Methylation | 1.27E-07 | 1.48E-10 |
| ALOX5 | merck2-NM_000698_at | 10q11.2 | Overexpression | 2.35E-03 | 8.73E-03 |
| AMD1 | merck-NM_001634_at | 6q21 |  | 1.22E-07 | 1.51E-04 |
| ANXA2 | merck2-AK092006_s_at | 15q22.2 |  | 2.45E-04 | 9.42E-02 |
| APC | merck-AJ707194_a_at | 5q21-q22 | Downregulation | 2.68E-05 | 1.91E-01 |
| AR | merck-T68445_a_at | Xq12 |  | 2.61E-08 | 8.00E-02 |
| ASPSCR1 | merck-NM_024083_at | 17q25.3 | Translocation | 2.18E-08 | 2.36E-02 |
| AURKA | merck-NM_198436_s_at | 20q13 | Overexpression | 1.30E-06 | 2.38E-07 |
| AURKB | merck-NM_004217_at | 17p13.1 | Overexpression | 8.63E-10 | 1.69E-02 |
| AXL | merck-NM_001699_at | 19q13.1 | Overexpression | 2.53E-04 | 6.16E-03 |
| BCL2 | merck-NM_000633_at | 18q21.3 | Mutation | 2.36E-05 | 4.89E-03 |
| BHMT | merck-NM_001713_at | 5q14.1 | Downregulation | 6.80E-05 | 9.40E-02 |
| BIRC5 | merck-ENST00000333706_x_at | 17q25 |  | 1.06E-08 | 1.70E-03 |
| BRAF | merck-AK098095_s_at | 7q34 |  | 3.57E-06 | 3.72E-01 |
| BTG3 | merck-NM_006806_s_at | 21q21.1 | Methylation | 1.18E-04 | 4.90E-02 |
| C3 | merck-NM_000064_at | 19p13.3-p13.2 | Overexpression | 5.23E-02 | 5.90E-03 |
| CCND1 | merck-NM_053056_at | 11q13 | Unclassified | 1.23E-03 | 9.04E-03 |
| CCNE1 | merck-NM_001238_at | 19q12 | Overexpression | 8.01E-10 | 1.53E-04 |
| CD151 | merck-NM_139029_at | 11p15.5 | Overexpression | 9.46E-02 | 1.64E-02 |
| CDH1 | merck-NM_004360_at | 16q22.1 | Methylation | 5.77E-03 | 1.54E-02 |
| CDKN2A | merck2-U38945_at | 9p21 | Methylation | 1.02E-02 | 1.26E-01 |
| CDKN2B | merck-NM_078487_at | 9p21 | Methylation | 2.04E-03 | 1.20E-03 |
| CEP290 | merck-NM_025114_s_at | 12q21.32 |  | 5.99E-07 | 4.55E-04 |
| CORO6 | merck2-AL833952_at | 17q11.2 | Methylation | 6.01E-08 | 1.08E-01 |
| CTDSPL | merck-NM_005808_at | 3p21.3 | Mutation | 3.15E-06 | 2.61E-05 |
| CXCR4 | merck-NM_003467_at | 2q21 | Overexpression | 6.87E-02 | 4.13E-01 |
| CYBA | merck2-NM_000101_at | 16q24 | Overexpression | 3.91E-06 | 4.26E-04 |
| ENO2 | merck-NM_001975_at | 12p13 | Overexpression | 1.99E-05 | 6.17E-04 |
| ESR1 | merck-NM_000125_at | 6q25.1 | Mutation | 2.64E-01 | 2.99E-04 |
| ETS1 | merck-NM_005238_at | 11q23.3 | Overexpression | 2.52E-03 | 1.56E-10 |
| FAM107A | merck-NM_007177_at | 3p21.1 | Methylation | 1.88E-02 | 3.94E-04 |
| FNIP2 | merck-AK057981_at | 4q32.1 | Downregulation | 7.42E-08 | 3.54E-01 |
| FTL | merck2-ENST00000305989_at | 19q13.33 | Overexpression | 2.76E-02 | 2.35E-01 |
| FZD4 | merck-NM_012193_at | 11q14.2 |  | 5.82E-07 | 3.84E-05 |
| GAPDH | AFFX-HUMGAPDH/M33197_3_at | 12p13 |  | 3.21E-05 | 4.92E-09 |
| GNL3 | merck-NM_206826_at | 3p21.1 | Unclassified | 7.73E-07 | 2.18E-02 |
| HDAC1 | merck-NM_004964_at | 1p34 |  | 8.74E-03 | 4.06E-04 |
| HLF | merck2-BC036093_at | 17q22 |  | 5.37E-05 | 7.17E-04 |
| HPX | merck2-BC005395_at | 11p15.5-p15.4 | Overexpression | 1.02E-07 | 8.75E-01 |
| HSPB1 | merck2-NM_001540_at | 7q11.23 | Overexpression | 3.29E-04 | 7.91E-02 |
| IGF1R | merck-ENST00000333402_s_at | 15q26.3 | Overexpression | 1.66E-04 | 7.09E-02 |
| ITGA6 | merck2-BX537483_at | 2q31.1 |  | 1.18E-09 | 4.22E-06 |
| JAG1 | merck2-BC098393_at | 20p12.1-p11.23 |  | 3.90E-03 | 1.45E-06 |
| JAK1 | merck-NM_002227_at | 1p32.3-p31.3 | Overexpression | 3.58E-08 | 4.63E-02 |
| JUP | merck-NM_002230_s_at | 17q21 | Methylation | 4.94E-03 | 2.92E-04 |
| KDR | merck-NM_002253_at | 4q11-q12 |  | 5.34E-06 | 1.29E-11 |
| KLF4 | merck2-AK095134_at | 9q31 |  | 1.08E-04 | 2.03E-02 |
| MANF | merck-NM_006010_at | 3p21.1 | Mutation | 1.99E-05 | 7.29E-03 |
| MKI67 | merck-BI868409_a_at | 10q26.2 | Overexpression | 1.29E-03 | 2.24E-05 |
| MMP3 | merck-NM_002422_at | 11q22.3 | Mutation | 2.51E-08 | 2.27E-02 |
| MMP9 | merck2-NM_004994_at | 20q11.2-q13.1 |  | 2.44E-04 | 1.78E-02 |
| MPZL2 | merck-NM_005797_at | 11q24 |  | 1.56E-05 | 6.26E-07 |
| MSTO1 | merck2-BC002535_a_at | 1q22 | Overexpression | 1.32E-12 | 4.38E-02 |
| MYCN | merck-NM_005378_s_at | 2p24.3 |  | 2.49E-03 | 1.27E-07 |
| MYO6 | merck-DR761543_a_at | 6q13 |  | 1.53E-07 | 4.52E-02 |
| PLOD2 | merck-NM_000935_at | 3q24 |  | 4.27E-04 | 2.31E-10 |
| PPIA | merck2-BC005982_x_at | 7p13 | Overexpression | 1.14E-03 | 1.15E-03 |
| PRDX6 | merck-CR598010_a_at | 1q25.1 | Overexpression | 1.83E-02 | 1.91E-02 |
| RAD23B | merck-AK125226_a_at | 9q31.2 | Overexpression | 1.82E-03 | 1.74E-02 |
| RAPGEF5 | merck-BC039203_at | 7p15.3 |  | 3.87E-04 | 2.42E-07 |
| ROR1 | merck-NM_005012_s_at | 1p32-p31 | Overexpression | 1.58E-05 | 9.25E-01 |
| RPL13A | merck2-BG231246_x_at | 19q13.3 |  | 1.36E-03 | 6.40E-01 |
| SAA1 | merck2-NM_030754_s_at | 11p15.1 | Overexpression | 1.64E-08 | 6.24E-10 |
| SLC22A2 | merck2-X98333_at | 6q25.3 |  | 4.56E-07 | 4.88E-03 |
| SLC5A8 | merck-BG570430_a_at | 12q23.1 |  | 5.84E-05 | 2.95E-04 |
| SLC6A3 | merck-NM_001044_at | 5p15.3 |  | 2.04E-02 | 2.32E-02 |
| SMAD4 | merck-NM_005359_at | 18q21.1 |  | 8.93E-03 | 5.03E-03 |
| SOCS3 | merck-NM_003955_at | 17q25.3 | Unclassified | 2.46E-04 | 9.79E-02 |
| SOD2 | merck-BC001980_s_at | 6q25.3 | Overexpression | 7.71E-03 | 5.30E-05 |
| SPINK1 | merck-NM_003122_s_at | 5q32 |  | 2.28E-04 | 1.35E-04 |
| SPRY1 | merck-NM_199327_s_at | 4q28.1 |  | 4.13E-03 | 2.43E-09 |
| TEK | merck-NM_000459_at | 9p21 | Downregulation | 6.06E-07 | 3.75E-10 |
| TFAP2A | merck-ENST00000379613_at | 6p24 | Overexpression | 4.45E-10 | 1.94E-02 |
| TGFBR3 | merck-ENST00000370399_s_at | 1p33-p32 | Downregulation | 1.31E-03 | 6.52E-03 |
| TGM2 | merck-NM_004613_at | 20q12 | Overexpression | 1.40E-03 | 2.41E-01 |
| THRB | merck2-NM_000461_at | 3p24.2 | Downregulation | 6.12E-06 | 1.84E-02 |
| TOP2A | merck-AK024080_a_at | 17q21-q22 | Overexpression | 7.43E-05 | 1.19E-05 |
| TPPP | merck2-AB017016_at | 5p15.3 | Unclassified | 3.84E-03 | 2.51E-02 |
| UBE2S | merck2-BI602361_s_at | 19q13.43 | Overexpression | 1.51E-08 | 4.45E-04 |
| WASF2 | merck-CR597572_at | 1p36.11 | Overexpression | 2.99E-04 | 3.20E-03 |
